# Investigating the Behavior of Thin-Film Formation over Time as a Function of Precursor Concentration and Gas Residence Time in Nitrogen Dielectric Barrier Discharge

**DOI:** 10.3390/ma17040875

**Published:** 2024-02-14

**Authors:** Faegheh Fotouhiardakani, Alex Destrieux, Jacopo Profili, Morgane Laurent, Sethumadhavan Ravichandran, Gowri Dorairaju, Gaetan Laroche

**Affiliations:** 1 Laboratoire d’Ingénierie de Surface, Centre de Recherche sur les Matériaux Avancés, Département de Génie des Mines, de la Métallurgie et des Matériaux, Université Laval, Quebec, QC G1V 0A6, Canada; faegheh.fotouhi-ardakani.1@ulaval.ca (F.F.); alex.destrieux.1@ulaval.ca (A.D.); profili.jacopo@gmail.com (J.P.); 2 Centre de Recherche du CHU de Québec, Hôpital St François d’Assise, 10 Rue de L’Espinay, Québec, QC G1L 3L5, Canada; 3 Saint-Gobain Research North America, 9 Goddard Rd, Northborough, MA 01532, USAsethumadhavan.ravichandran@saint-gobain.com (S.R.); gowri.dorairaju@saint-gobain.com (G.D.)

**Keywords:** plasma, fluoropolymer, surface treatment, hydrophilicity, coating, dielectric barrier discharge

## Abstract

This study aims to establish a correlation between the fragmentation process and the growth mechanisms of a coating deposited on a fluoropolymer. Deposition was carried out using dielectric barrier discharge at atmospheric pressure, employing an oxygen-containing organic precursor in a nitrogen environment. The findings reveal that the impact of precursor concentration on the formation of specific functionalities is more significant than the influence of treatment time. The X-ray photoelectron spectroscopy (XPS) results obtained indicate a reduction in the N/O ratio on the coating’s surface as the precursor concentration in the discharge increases. Fourier transform infrared spectroscopy (FTIR) analysis, conducted in the spectral range of 1500 cm^−1^ to 1800 cm^−1^, confirmed the connection between the chemical properties of plasma-deposited thin films and the concentration of organic precursors in the discharge. Furthermore, the emergence of nitrile moieties (C≡N) in the FTIR spectrum at 2160 cm^−1^ was noted under specific experimental conditions.

## 1. Introduction

Significant advancements have been achieved in the synthesis of nanometric organic thin films due to their diverse applications in the electrical and optical fields involving adhesive and composite materials [1,2,3,4]. Indeed, thin coatings offer the advantage of modifying surface properties without impacting the bulk material’s characteristics [5]. Over the past decade, atmospheric pressure plasma (APP) has gained popularity as an increasingly favored tool for producing advanced thin coatings given its beneficial qualities such as high speed, cost-effectiveness, and minimal chemical waste generation [6,7,8,9,10]. Many APP techniques benefit from the ability to move a substrate under relatively small plasma sources with minimal operational effort, facilitating the treatment of large areas [11]. However, the behavior of plasma at atmospheric pressure remains poorly understood due to the presence of various species in the gas phase that influence both the discharge and the coating [11,12].

Furthermore, the production of thin-film coatings containing functional groups such as alcohol [13], carboxyl [14], thiol [15], aldehyde [16], and epoxy [17] necessitates the use of an organic precursor with specific chemical functionalities in addition to a carrier gas. Typically, these specific functional groups, which include nitrogen- and oxygen-containing polar groups, can enhance the long-term wettability of surfaces that are inherently hydrophobic before plasma coating [18,19]. Similarly, introducing only a non-polymerizable gas within the discharge usually results in a simple functionalization of the first atomic layer, leading to short-term hydrophilicity enhancement [11,20]. However, combining a non-polymerized oxygen-containing precursor with nitrogen as a carrier gas has the potential to generate the mentioned chemical functionalities. This is because nitrogen is likely to react with the precursor, incorporating it within the coating and enhancing long-term wettability [21].

Experimental parameters, including power, precursor concentration, flow rate, gas residence time, and treatment time, can influence the chemical and physical behavior of gases/vapors injected into a plasma as well as the growth mode of thin films [11,22]. Therefore, it is essential to determine the optimal parameters for a specific system to control the fragmentation and formation of the required functional groups, as these parameters may not work uniformly for all systems [23].

Recently, several studies have focused on the soft polymerization of precursors, characterized by minimizing monomer fragmentation and the integration of carrier gas atoms within the deposited coating. Under these conditions, the structures of the precursor molecules are mostly preserved as the repeating unit of the plasma polymer [24,25,26]. Although the term “monomer” is commonly associated with polymerized gases, it can also be applied to non-polymerized gases, particularly in the context of soft polymerization. Moreover, the reactions induced by the exposure of molecules to plasma are influenced by the energy transferred to them and the plasma discharge. In this case, the term “soft plasma polymerization regime” was initially used to describe plasma processes with low energy input [27], but it has now been expanded to include all plasma process conditions that enable controlled polymerization of precursors while selectively activating polymerization sites and preserving the formation of other chemical functionalities of the monomers. This regime is typically characterized by the amount of energy per monomer molecule [27,28].

Some studies suggest that pulsed power plasma minimizes fragmentation and the incorporation of the carrier gas into precursor molecules [29,30]. In this case, low duty cycles (e.g., with a higher period of time wherein plasma is turned off compared to the one wherein it is turned on) prioritize soft polymerization processes [11]. It is important to note that, at atmospheric pressure, monomer transport is advection-driven. Therefore, gas flow patterns, velocity, and gas residence time (Ƭr) are particularly important for controlling fragmentation [31]. Ƭr represents the average time that a gas stays in a discharge, measured by the size of the plasma region between the dielectric barrier discharge (DBD) electrodes and the gas flow rate [26,32]. Additionally, the stability of the process over time is not fully understood, especially when the gas residence time varies with different total flows [33,34].

In this context, the aim of this study was to deposit a thin film on ethylene tetrafluoroethylene (ETFE) surfaces under atmospheric pressure. A model ketone precursor molecule [35,36], which was otherwise nitrogen-free, was vaporized between the electrodes along with the carrier gas. The resulting plasma coatings contained functionalities such as C=O, C=O–N, C–C, and C≡N. To analyze the chemistry and growth rate of the coating in relation to the total flow, precursor concentration, and precursor residence time, X-ray photoelectron spectroscopy (XPS), Fourier-transform infrared spectroscopy (FTIR), and profilometry were employed.

## 2. Materials and Methods

### 2.1. Materials

Films of ETFE with a thickness of 0.127 mm (provided by Saint-Gobain Research North America, Northborough, MA, USA) underwent plasma treatment in static mode for various durations, ranging from 1 to 7 min, in a DBD system. Nitrogen (99.999%; AirLiquide, Montreal, QC, Canada) served as the carrier gas, and a model ketone molecule, devoid of nitrogen and selected from a group of precursors previously disclosed by Bravet et al. and Kowalewski et al. [35,36], was introduced into the plasma region concurrently with the carrier gas. The selection of this organic precursor was based on its capacity to generate polar compounds with nitrogen during plasma discharge. The polymer film was positioned on the ground electrode in the reactor without any preceding cleaning or preparation processes.

### 2.2. Experimental Setup and Conditions

The atmospheric pressure dielectric barrier discharge system employed in this study has been previously detailed [7,8]. In brief, the experimental setup consisted of a generator, a transformer, two flat stainless-steel high-voltage (HV) electrodes arranged parallel to a cylindrical ground electrode, and a vacuum system. These electrodes were positioned within a stainless-steel chamber. Each high-voltage electrode had a surface area of 0.95 cm × 14.9 cm, and the ground electrode had a diameter of 150 mm. Polymer films were situated on the surface of the ground electrode, parallel to the high-voltage electrodes.

The discharge power supply was constructed using an arbitrary function generator (AFG-2021, Tektronix, Beaverton, OR, USA) connected to an audio amplifier PL380 obtained from QSC Audio Products, LLC (Costa Mesa, CA, USA), operating in the 10–100 kHz range. A transformer (RAFTabtronics) amplified the signal, providing an applied voltage (V_a_) of up to 18 kV between the HV and ground electrodes, with a bandwidth of 5 to 15 kHz. The sinusoidal voltage used in this study had a frequency of 5 kHz. The duty cycle (DC) employed to control the power during treatment was set to 30%, and the power over 10 ms (the chosen time for the duty cycle interval) was 0.75 W·cm^−2^.

The total gas flow (N_2_ + precursor) was adjusted within the range of 1 to 5 SLM (Standard Liter per Minute), and the interelectrode gap was set to 1 mm. The precursor amount varied between 1% and 40% with respect to the amount of N_2_.

### 2.3. Characterization Techniques

#### 2.3.1. Electrical Characterization

The applied voltage was measured at the high-voltage transformer output using a P6015A high-voltage probe from Tektronix (bandwidth 75 MHz, Beaverton, OR, USA). Current measurements were taken with a Pearson 4100 current monitor located on the ground side outside the reactor (10 ns rise time, bandwidth: 140 Hz–35 MHz). Additionally, the charge was measured by incorporating a 100 nF capacitor in series with the discharge cell on the ground side of the discharge. The voltage across the capacitor was monitored using a TPP0500B passive voltage probe obtained from Tektronix (bandwidth 500 MHz). Subsequently, all probes were connected to a MDO3054 oscilloscope obtained from Tektronix (bandwidth: 500 MHz, 8-bit vertical resolution). It is worth noting that the power was maintained at a constant level and calculated as follows:

$$\bar{P}=\frac{1}{T}\int_{0}^{T}V_{a}\left(t\right)\cdot C_{m}\frac{dV_{m}\left(t\right)}{dt}dt=\frac{1}{T}\int_{0}^{T}V_{a}\left(t\right)\cdot C_{m}dV_{m}(t)=f\oint V_{a}dQ_{m}$$

Here, V_a_ represents the applied voltage, C_m_ is the measurement capacitor, V_m_ denotes the measured voltage across C_m_, and f stands for the frequency of the applied voltage. This calculation corresponds to the power over one period. The power over the 10 ms burst (0.75 W·cm^−2^) was determined as follows:

$$P_{burst}=DC(\%)\times \bar{P}.$$

#### 2.3.2. X-ray Photoelectron Spectroscopy

XPS (PHI 5600-ci spectrometer, Physical Electronics, Chanhassen, MN, USA) was used to measure the relative atomic composition on the various investigated surfaces. The measurements were carried out with an incident detection angle of 45° on sample areas of 0.5 mm^2^ at a residual pressure of 8 × 10^−6^ Pa. A survey spectrum (0–1200 eV) was first recorded using a standard aluminum K_α_ X-ray source (1488.6 eV) operated at 300 W with a charge neutralizer. A monochromatic magnesium anode (1253.6 eV) was utilized at 150 W without charge neutralization for high-resolution carbon spectra (280–300 eV). The CF_2_ component of the C1s spectrum (291.0 eV) was utilized to calibrate the energy scale for ETFE, while the hydrocarbon component of the C1s spectrum (285.0 eV) was used for the coating. Multipak (9.0 V) software was employed to process the XPS survey scans. Multipak was used to curve fit high-resolution C1s peaks using Gaussian–Lorentzian peak forms over an iterated Shirley background, with the full width at half maximum (FWHM) of each line shape kept between 0.9 and 1.2 eV. Each sample was subjected to four measurements. Furthermore, this coating investigation was performed twice for each plasma setting to ensure that the process was repeatable.

#### 2.3.3. Attenuated Total Reflectance Fourier Transform Infrared Spectroscopy (ATR-FTIR)

The chemical functionalities of the coating were identified using Fourier-transform infrared spectroscopy in attenuated total reflection mode (FTIR-ATR). Spectra were recorded on a Cary 660 Series FTIR spectrometer with a resolution of 4 cm^−1^ (Agilent, Mulgrave, Australia), accumulating 128 scans between 4000 cm^−1^ and 400 cm^−1^. A Harrick SplitPea™ (Pleasantville, NY, USA) was configured to ensure contact between the sample and the silicon-based ATR crystal. For each sample, measurements were taken at four different points on the coating. These values were used to calculate the standard deviation of the average area under the peaks corresponding to chemical moieties of interest for the growth mode study.

Peak curve fitting in the region between 1500 and 1800 cm^−1^ was performed using OriginPro software version 2021 via a Voigt-shaped curve-fitting method. This region exhibits features corresponding to two C=O bonds involved in different chemical moieties, namely, amide (N–RC=O) and ketone (R_2_C=O). This fitting method suggests using an additional band at 1620 cm^−1^ to fit the curve of this region. According to the literature, this band can be assigned to amine groups (N–H) in the deposited films, as derivatization experiments with chlorobenzaldehyde [37] (not shown) resulted in the disappearance of this feature [38].

#### 2.3.4. Profilometry

Coating thickness was determined using a surface profiler (Dektak 150, Veeco, Plainview, NY, USA) equipped with a 2.5 µm stylus and possessing an applied weight of 0.3 mg. This mechanical approach requires the presence of a step between the substrate surface and that of the plasma-deposited film. These steps were developed by covering part of the substrate with a very thin film of ETFE during the plasma process. It is essential to note that this covering was extremely thin and placed in close contact with the substrate to minimize shadowing and material accumulation near the step edge. The height of the resulting step was determined by longitudinally displacing the stylus from the plasma-deposited film to the uncoated polymer sample [39,40]. To ensure accuracy, a minimum of eight measurements were taken per sample to obtain a reliable standard deviation.

## 3. Results

### 3.1. Electrical Characterization

**Figure 1:**
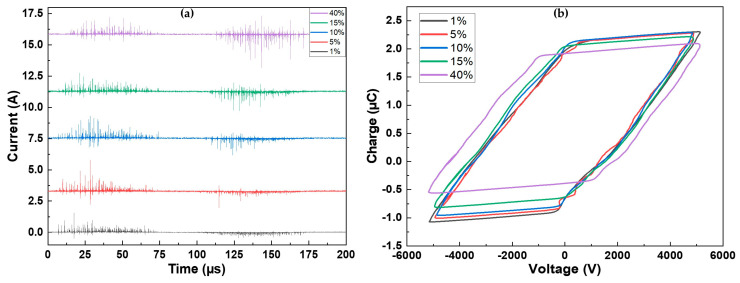
(**a**) Current over one period for the different precursor concentrations; (**b**) corresponding Lissajous figures.

Figure 1a illustrates the current measured over one period of applied voltage for all the investigated precursor concentrations. Regardless of the concentration, the discharge is characterized by short current pulses, indicating a filamentary discharge regime [41]. The discharge exhibits similarity on both alternances of the voltage (positive: 0–100 μs; negative: 100–200 μs). However, a slight modification of the current on the negative alternance is evident with an increase in precursor concentration. At low precursor concentrations, the observed current spikes have low amplitudes (<0.5 A). As the precursor amount rises and reaches 40%, the amplitude of the current spikes also increases, reaching a maximum of 2.6 A. The injection of a precursor is known to destabilize the discharge and is associated with discharge filamentation [42].

As depicted in Figure 1b, not many changes were observed in the Lissajous figures (Q-V plots) recorded for various precursor concentrations, as their shapes are similar for all conditions (further explanations are provided in [43]). The main difference lies in the amplitude of the charge, which decreased as the precursor concentration increased. For precursor concentrations ranging between 1% and 15%, the behavior was similar. For the highest precursor amount, the width of the figure increased, and the charge amplitude was the lowest. It is noteworthy that the measured power is the same for all conditions $\bar{P}\approx 2.7\;{W\cdot cm}^{-2}$. Breakdown also occurs slightly later for 40% of precursor. This is evident from the time shift observed in the appearance of the first filaments in Figure 1a, occurring approximately 7 µs later at 40% compared to 1%, as well as from the voltage at which the breakdown is observed ($V_{a,pp}\approx -330\;V$ at 1% vs. $V_{a,pp}\approx 1000\;V$ at 40%).

### 3.2. Elemental Composition of the Surface for 1 SLM Total Flow

**Figure 2:**
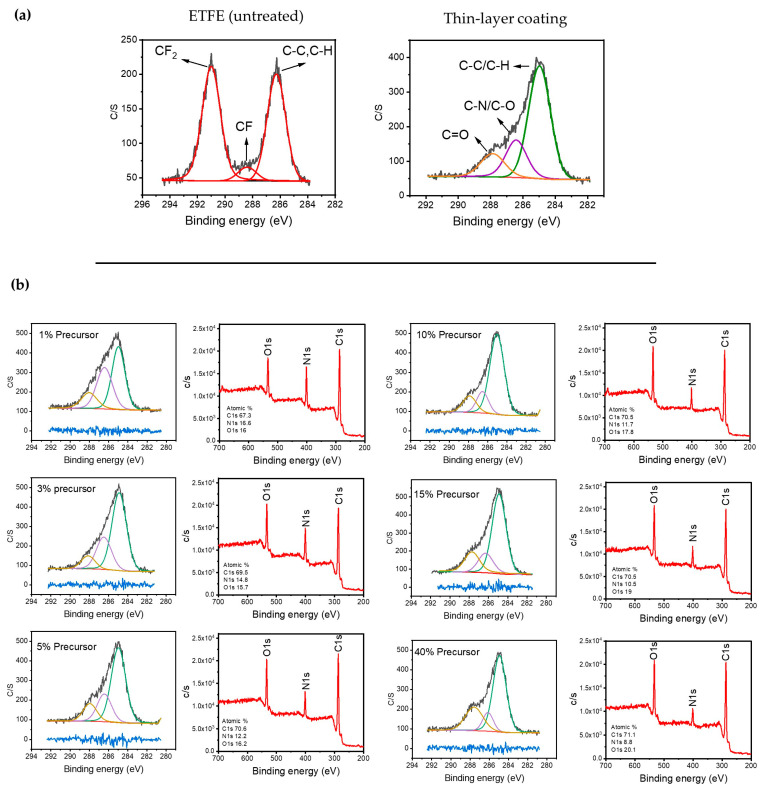
(**a**) Typical C1s XPS spectra of the untreated ETFE and the surfaces after a long plasma treatment; (**b**) evolution of the survey and C1s XPS spectra as a function of the precursor concentration after 1 min of plasma treatment.

Figure 2a displays the high-resolution C1s XPS spectrum of the ETFE polymer surface before and after plasma treatment. The spectrum of the untreated surface structure can be divided into three bands assigned to CF_2_ (291 eV), C–F (288.4 eV), and single-bonded C–C/C–H-containing species (286.5 eV) [44,45]. Following plasma deposition for several minutes using 1 SLM total flow containing 1% precursor, the new C1s spectrum of the treated surface could also be fitted using three bands. The band at 285 eV was attributed to C–C and C–H, while those at 286.5 eV and 288 eV were assigned to hydrophilic single-bonded C–N/C–O and C=O, respectively [21,46,47]. Importantly, both C–N and C–O formed because of the fragmentation of the precursor molecule along with the incorporation of the carrier gas (N_2_) within the chemical structure of the plasma-deposited coating.

The apparent shift of the entire C1s spectrum for the plasma-treated polymer with respect to that of the pristine sample, along with the absence of the CF_2_ feature at 291 eV, supports the deposition of a coating on the ETFE with a thickness greater than 5–10 nm, which is the maximal depth of analysis of the XPS technique for organic materials. Figure 2b illustrates the typical C1s spectra for coatings deposited using various precursor concentrations ranging from 1 to 40% with a 1 SLM total flow (1 min of treatment) along with the survey spectra of each condition.

The most noticeable difference between these C1s spectra is that the relative intensity of the 287.8 eV band assigned to the oxygen group (C=O) increased with the precursor concentration. This increase was observed with a reduction in the band attributed to C-N/C-O. Additionally, a comparison of the survey spectra reveals a significantly higher intensity for the nitrogen peak at low precursor concentrations, which decreases as the precursor concentration increases in the discharge. This suggests that a soft polymerization (i.e., radical polymerization) is likely favored compared to crosslinking and nitrogen incorporation in the coating for higher precursor concentrations [48].

**Figure 3:**
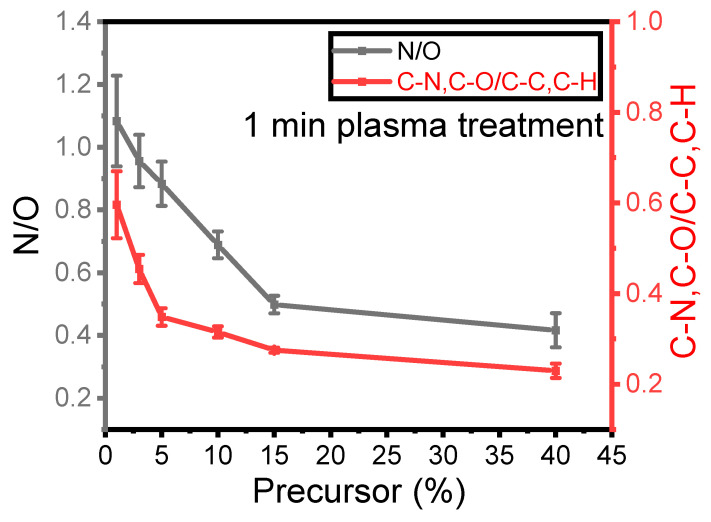
Evolution of the XPS N/O and C–N, C–O/C–C, and C–H ratios as a function of the precursor concentration for a 1 min plasma treatment.

Figure 3 illustrates the nitrogen-to-oxygen ratio (N/O) measured from the survey spectra in the plasma coating as a function of precursor concentration for a 1 SLM total flow after 1 min of treatment. As mentioned earlier, nitrogen originates from the reactive gas, while oxygen comes from the precursor. The ratio between these two elements indicates the impact of precursor concentration on the deposited elements on the surface under a constant total flow with relatively low differences in the nitrogen flow within the plasma discharge. This ratio can also be compared to the ratio of C–N and C–O to C–C and C–H as a function of precursor concentration, as C–C and C–H originate from the precursor structure, while C–N and C–O moieties are formed because of the fragmentation and new bond formation process.

The N/O ratio on the surface decreased as the precursor concentration increased. Specifically, the N/O ratio decreased from 1.2 to 0.5 with an increase in concentration from 1% to 15%, with a further smaller reduction to 0.4 when the precursor content inside the discharge increased to 40%. Similarly, the C–N and C–O (at 286.5 eV) to C–C and C–H (at 285 eV) ratio is plotted in the same Figure 3 to better understand how nitrogen and oxygen are incorporated into the thin-film surface. As observed for the N/O ratio, the ratio from the high-resolution spectra exhibits a very similar trend as a function of precursor concentration. This value decreased from 0.6 to 0.2 when the concentration increased from 1% to 40%. It can be concluded that for a given total flow rate, the amount of nitrogen in the coating and the formation of C–N/C–O depend on the precursor concentration inside the discharge and hence on the energy per precursor molecule that is available.

Comparing these two ratios confirms an inverse relationship between the integration of nitrogen into the coating and the amount of precursor injected into the discharge. These results suggest that when the quantity of precursor molecules ([M], in g·h^−1^) in the discharge is lowered at constant power (P, in Watt), more energy is available for each molecule (E_D_ = P/[M]) [28]. Therefore, higher E_D_ results in more efficient chemical reactions between the carrier gas and the precursor molecules. This observation is more evident for a value of [M] lower than 15%, as such an amount seems to be already high with respect to the energy available for promoting efficient chemical reactions.

### 3.3. Evolution of the Coating Chemical Composition as a Function of Time and Total Flow

**Figure 4:**
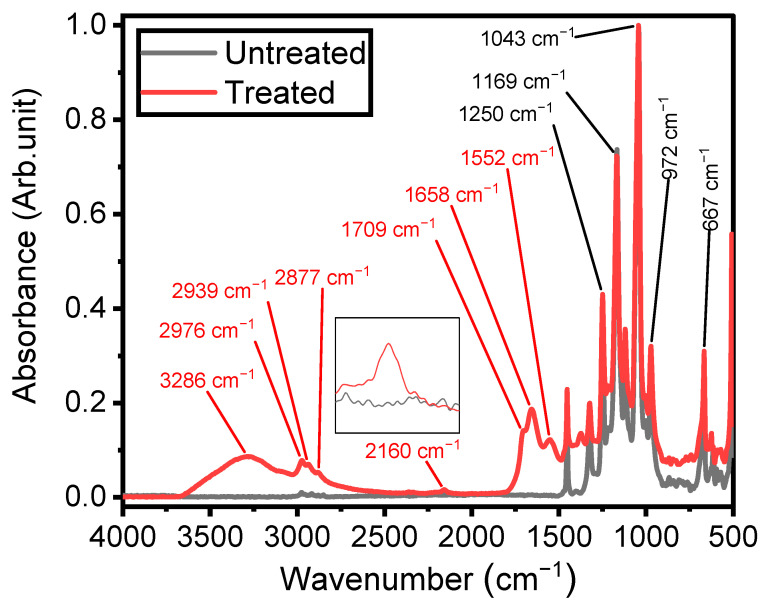
Fourier-transform infrared spectra of untreated ETFE and plasma-treated ETFE samples after 3 min using 1 SLM total flow containing 1% precursor.

IR spectroscopy was employed to investigate the chemistry and structures of plasma-coated ETFE surfaces, providing insights into the mechanisms underlying thin-film growth [18]. As depicted in Figure 4, the spectrum of the ETFE film consists of several strong bands with features assigned to stretching mode vibrations of CF and CF_2_ groups, primarily located between 500 cm^−1^ and 1500 cm^−1^. Bands with lower absorbances can also be observed between 2800 cm^−1^ and 3000 cm^−1^, corresponding to stretching modes of CH_2_ and CH_3_ groups [49].

Figure 4 also illustrates the spectrum of the plasma-deposited coating after 3 min of plasma treatment using a 1 SLM total flow containing 1% organic precursor, revealing new infrared features characteristic of its chemical structure. Considering that the depth of an ATR-FTIR investigation using a silicon crystal at an angle of incidence of 45° is typically 2–3 µm between 1000 and 1400 cm^−1^, the presence of ETFE spectral components in this spectral region after plasma treatment clearly indicates that the thickness of the coatings was less than 2 µm. The existence of a plasma-deposited coating is emphasized by the strong band at 3300 cm^−1^, attributed to the stretching mode vibration of O–H and/or N–H groups [21,38,50]. Additionally, the increased intensity of the features in the 2800–3000 cm^−1^ spectral range is a clear indication of the formation of a hydrocarbon structure upon plasma treatment [18].

Under certain plasma operating conditions, the stretching mode vibration of C≡N in nitrile groups appears at 2160 cm^−1^ (Figure 4) [29]. Other studies have also reported this band, including some instances obtained in a hydrocarbon precursor/N_2_ plasma environment [51,52]. Features attributed to ketone, amide, and amine, expected to be present in the plasma-deposited film, were observed between 1500 and 1800 cm^−1^. The presence of N-H bonds in the amide group is confirmed by the absorption at 1552 cm^−1^, assigned to the bending mode of this chemical group [29]. The coatings exhibited at least two features related to the hydrophilic C=O group. The first one, observed as a shoulder at 1709 cm^−1^, was attributed to C=O bonds in ketone functionalities (RC(=O) R′) [18], associated with the chemical structure of the ketone model precursor molecule resulting from the soft polymerization process. The second, at 1658 cm^−1^, can be associated with amide functionalities (N–C=O) [18,21,53], linked to stronger precursor reactivity (Figure 4).

**Figure 5:**
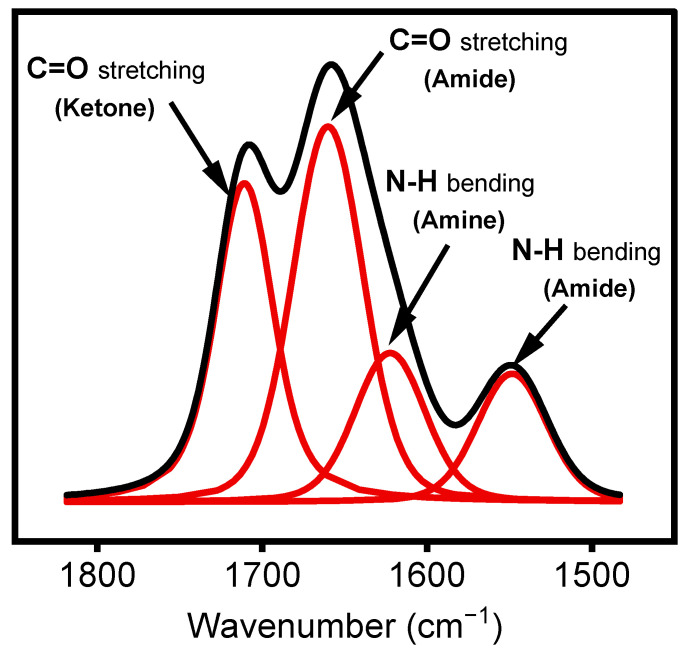
Curve fitting (red lines) of FTIR spectrum (Black lines) in the region between 1500 cm^−1^ and 1800 cm^−1^.

As shown in Figure 5, the integrated peak area ratio of the features at 1658 cm^−1^ and 1709 cm^−1^ (A_1658_/A_1709_) can be used to follow the organic precursor fragmentation as a function of the experimental parameters used during treatment. In the present study, the precursors varied between 1.2 and 48 g·h^−1^ for concentrations ranging from 1% to 40% for a given 1 SLM total flow, as shown in Table 1.

**Figure 6:**
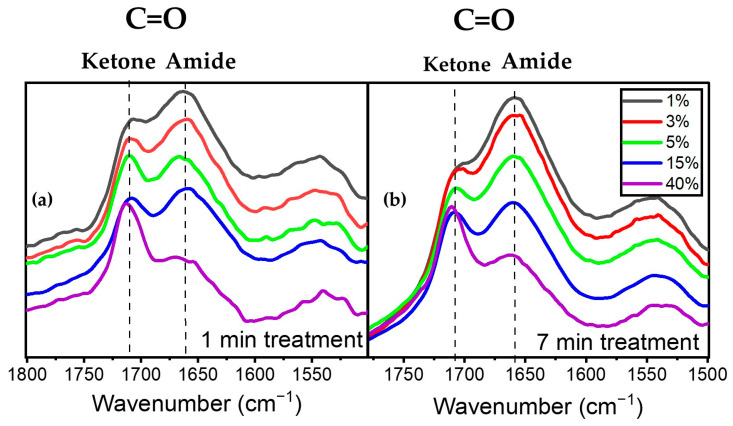
Evolution of the C=O amide and ketone as a function of precursor concentration (**a**) after 1 min of plasma treatment and (**b**) after 7 min of plasma treatment.

Figure 6a illustrates the evolution of the peaks at 1658 cm^−1^ assigned to amide (a nitrogen-containing compound) and 1709 cm^−1^ assigned to ketone for various precursor concentrations at 1 SLM total flow. An analysis of these spectral features after one minute of plasma treatment revealed that an increase in precursor concentration from 1% to 40% led to a decrease in the intensity of the amide peak at 1658 cm^−1^ relative to that of the 1709 cm^−1^ peak. This observation aligns with the decreasing trend of the N/O ratio derived from XPS analysis as a function of precursor concentration for a given total gas flow (Figure 3). A similar behavior in terms of the relative variation in the 1658 cm^−1^ and 1709 cm^−1^ features after 7 min of plasma treatment is also depicted in Figure 6b, albeit with a slight modification of the overall spectrum envelopes.

**Figure 7:**
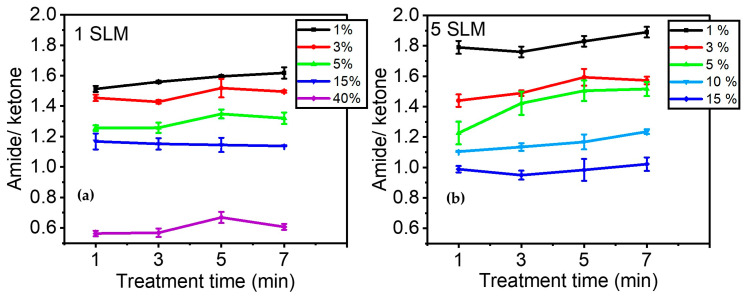
Evolution of the amide/ketone ratio as a function of precursor concentration over time (e.g., 1, 3, 5, and 7 min): (**a**) 1 SLM total flow (Ƭr: 8.5 ms); (**b**) 5 SLM total flow (Ƭr: 1.7 ms).

Figure 7a illustrates the evolution of A_1658_/A_1709_ as a function of precursor concentration after 1, 3, 5, and 7 min of plasma treatment. Overall, this figure demonstrates that the effect of precursor concentration is more significant than that of plasma treatment time in terms of the relative quantity of amide groups formed on the surface compared to other C=O-containing moieties. The ratio of amide/ketone decreased from ~1.6 to ~0.6 with an increase in precursor concentration from 1% to 40% in the discharge. On the other hand, a longer treatment time only slightly affected the number of amide groups in the coating. The most notable increase was observed for a precursor concentration of 1%, with A_1658_/A_1709_ changing from ~1.5 to ~1.6 between 1 and 7 min of treatment.

Figure 7b shows the evolution of A_1658_/A_1709_ when a total gas flow of 5 SLM was used to feed the discharge. As indicated in Table 1, the gas residence time decreased from 8.5 ms to 1.7 ms when the total flow increased from 1 SLM to 5 SLM. This implies that the behavior of molecular fragmentation as a function of precursor percentage was studied over a shorter gas residence time within the discharge. Essentially, a similar trend was observed between Figure 7a,b, with the relative concentration of amide/ketone being higher at low precursor concentrations. It is noteworthy that for low precursor concentrations (up to 5%), a higher gas flow (i.e., shorter gas residence time) led to a higher amide/ketone ratio, while it decreased for higher precursor concentrations (15%). Additionally, Figure 7b also aligns with the observation made in Figure 7a regarding the low impact of treatment time on the amide/ketone ratio, suggesting an almost constant plasma composition and, consequently, deposited coating chemistry for the investigated plasma duration times.

It is noteworthy that the behavior of the 1622 cm^−1^ feature, assigned to primary amine groups (Figure 5), with respect to the peak at 1709 cm^−1^ was also investigated, presenting the same trend as the amide band (results not shown). Accordingly, it can be concluded that both the formation of amide and amine proceeded simultaneously for all the investigated experimental conditions. As depicted in Figure 4, nitrile (C≡N) also formed during the plasma surface modification for some of the investigated experimental conditions, as highlighted by the appearance of an infrared peak centered at 2160 cm^−1^ [54]. This band was also observed in the work of Vallade and his colleagues [7] on plasma-treated ETFE in an N_2_/organic precursor environment using a DBD system and appeared under certain experimental conditions.

**Figure 8:**
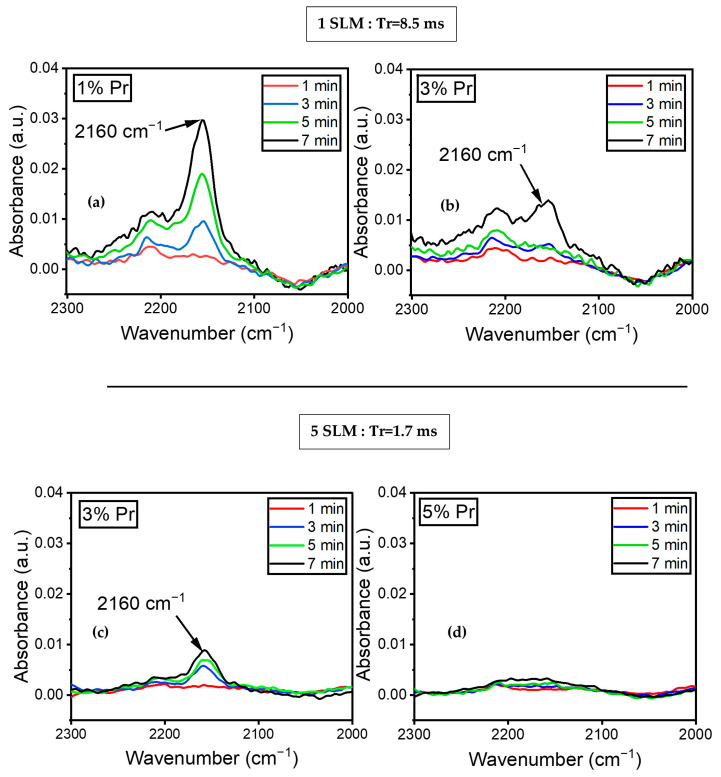
FTIR spectra of plasma-treated ETFE showing the evolution of the absorbance of the C≡N feature’s (2160 cm^−1^) evolution over time for (**a**) 1% precursor under a 1 SLM total flow; (**b**) 3% precursor under a 1 SLM total flow; (**c**) 3% precursor under a 5 SLM total flow; (**d**) 5% precursor under a 5 SLM total flow.

Figure 8a illustrates the variation in the absorbance of the peak at 2160 cm^−1^, assigned to the C≡N stretching mode, as a function of time (1, 3, 5, and 7 min) for a 1% precursor concentration under a 1 SLM total flow. The spectra did not show any peaks at 2160 cm^−1^ during the first minute of plasma treatment. Nitrile formation appeared to begin after 3 min of plasma treatment and continued to increase. Furthermore, Figure 8b demonstrates that with a 3% precursor in a 1 SLM total flow, the peak at 2160 cm^−1^ only appeared after seven minutes of plasma treatment. This indicates that both the concentration of the precursor and treatment time influence the formation of C≡N. Similar experiments were conducted with a total flow of 5 SLM to determine the influence of higher nitrogen content and shorter gas residence time in the discharge. Figure 8c illustrates the evolution of the peak at 2160 cm^−1^ using a 3% precursor under a 5 SLM total flow. The peak appeared after three minutes of treatment, with a slight increase in absorbance up to seven minutes of plasma treatment. Furthermore, Figure 8d shows no evidence of C≡N on the surface coating for a 5% precursor under a 5 SLM total flow. This data demonstrate that nitrile groups formed under low precursor concentrations either under a 1 or 5 SLM total flow (regardless of gas residence time), as more energy per molecule was available to form this highly energetic triple bond.

**Figure 9:**
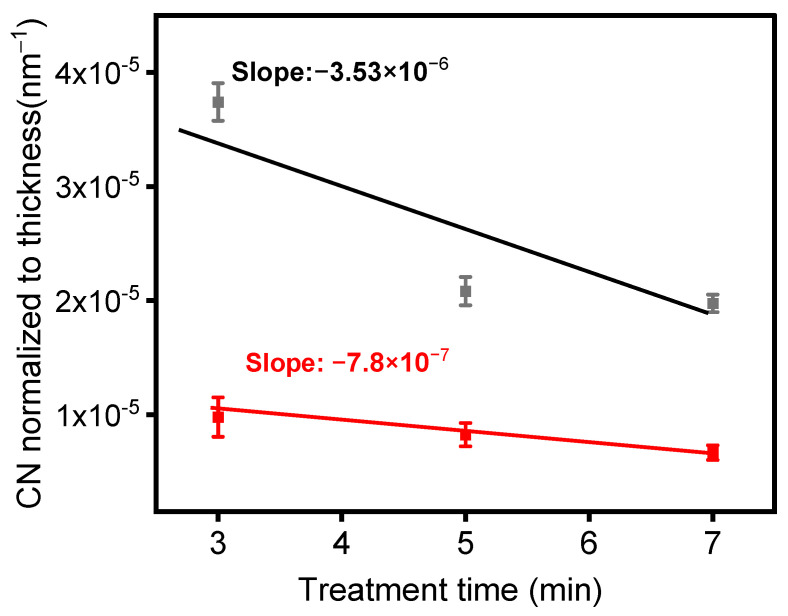
Normalized infrared absorbance of the C≡N intensity with respect to coating thickness as a function of treatment time for the following experimental conditions: black—1 SLM 1% precursor concentration (Ƭr = 8.5 ms); red—5 SLM-3% precursor concentration (Ƭr = 1.7 ms).

As shown in Figure 9, normalizing the peak absorbances of the spectra presented in Figure 8a,c relative to the film thicknesses enabled us to track the rate of formation of nitrile moieties under the experimental conditions used to generate these data. Two noteworthy behaviors are evident in Figure 9. Firstly, the quantity of CN groups in the deposited layer was higher at a lower total flow, indicating that a lower gas flow (or a higher gas residence time in the plasma) facilitated greater energy transfer to gas molecules within the plasma. Consequently, this led to a more efficient chemical reaction between the precursor and the carrier gas. Secondly, the CN group concentration in the layer visibly decreased with treatment time, signifying that the number of CN groups was higher near the ETFE interface, gradually diminishing as the deposited layer thickened. It is important to note that the rate of decay of the CN concentration with layer thickness was more pronounced at lower gas flow. Given the previous observation that the temperature of the electrodes significantly increased with treatment time [43], it is likely that the different types of decay observed in Figure 9 originate from the interaction between electrode temperature and gas flow. 

### 3.4. Coating Thickness and Growth Rate

**Figure 10:**
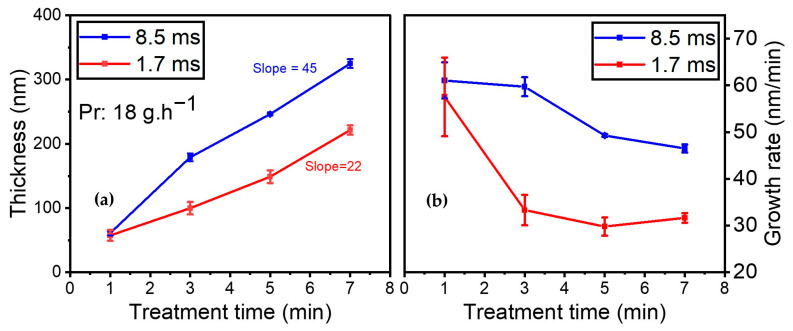
(**a**) Coating thickness (nm); (**b**) growth rate evolution (nm/min) for 18 g·h^−1^ precursor amount as a function of treatment time for the following residence times: blue—Ƭr = 8.5 ms (1 SLM total flow); red—Ƭr = 1.7 ms (5 SLM total flow).

It should be emphasized that both AFM and SEM images of the plasma-deposited coatings were recorded (results not shown). On the one hand, the AFM images reveal that the coating is homogeneous and closely matches the polymer topography. On the other hand, the SEM images indicate that the coating closely adapts to the underlying polymer, with the presence of holes measuring 200 nm, likely originating from the filamentary nature of the discharge. Additionally, the impact of gas residence time on coating thickness and growth rate was investigated. Figure 10a illustrates the development of the coating thickness while injecting 18 g·h^−1^ of the organic precursor into the discharge as a function of treatment time (i.e., 1, 3, 5, and 7 min) for 8.5 and 1.7 ms of gas residence time (with a total flow of 1 SLM and 5 SLM, respectively). Similar precursor amounts enable the same power per molecule under various gas residence time conditions.

As expected, the results indicate that the thickness increased with gas residence time. However, in the first minute of deposition, similar thicknesses were obtained for both conditions, indicating that gas residence time had no effect on the initial layer being deposited. Upon subjecting the substrate to longer treatment times in the plasma zone, the coating thickness increased to a greater extent when the gas residence time was higher (8.5 vs. 1.7 ms).

The thickness of the thin film increased from 61 nm to 325 nm over time (from 1 to 7 min of plasma treatment) when the gas residence time was 8.5 ms. Under a shorter gas residence time of 1.7 ms, between 1 min and 7 min of plasma treatment, the coating thickness rose from 57 nm to 221 nm. As indicated by the slopes of the best linear fit through these data points, the coating thickness was nearly twice as high at 8.5 ms of gas residence time (slope: 45 nm/min) than it was for the experiment at 1.7 ms (slope: 27 nm/min).

Additionally, the growth rate was assessed as a function of treatment time for various gas residence times (see Figure 10b). The growth rate (in nm/min) is calculated by dividing coating thickness by treatment time. Both experimental conditions showed a rapid growth rate of 60 nm/min for the first minute, after which the growth rate decreased with time. However, the trends were different for each gas residence time.

For the experiments carried out at 8.5 ms of gas residence time, the growth rate remained almost identical at 60 nm/min between 1 and 3 min of plasma treatment. It then dropped to 49 nm/min after 5 min of plasma treatment and 46 nm/min after 7 min. For 1.7 ms of gas residence time, the growth rate dropped strongly from 61 to 33 nm/min between 1 and 3 min of plasma treatment. It then reached a plateau and remained almost constant between 3 and 7 min of plasma treatment. Overall, as expected, reducing the gas residence time resulted in a significantly slower growth rate.

**Figure 11:**
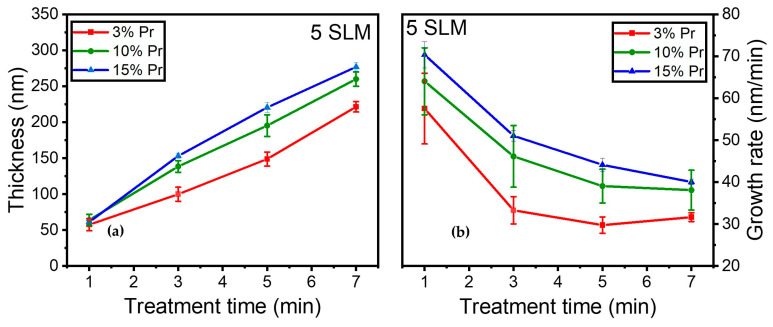
(**a**) Coating thickness (nm); (**b**) growth rate evolution (nm/min) as a function of treatment time for 3%, 10%, and 15% precursor under a 5 SLM total flow.

Moreover, the influence of precursor concentration on coating thickness and growth rate was investigated. Figure 11a illustrates the evolution of coating thickness for 3%, 10%, and 15% precursor concentrations as a function of various treatment times (i.e., 1, 3, 5, and 7 min) under a 5 SLM total flow (similar gas residence time).

The coating thickness increased steadily over time in all conditions, with notably thicker coatings observed at higher precursor concentrations. It is worth noting that a similar behavior was observed during the first minute of deposition, resulting in comparable coating thicknesses (≈65 nm) across different conditions.

Furthermore, Figure 11b shows that the growth rate displayed a trend comparable to that in Figure 10, exhibiting a rapid growth rate of 65 nm/min and reaching a plateau with longer treatment times. The growth rate decreased more significantly for the 3% precursor concentration between 1 and 3 min of treatment, stabilizing thereafter for durations longer than 3 min. In contrast, the 10% precursor exhibited a more gradual reduction in growth rate, stabilizing later, while the 15% precursor concentration demonstrated a gradual reduction in growth rate, starting from 65 nm/min and reaching 40 nm/min after 7 min of plasma treatment.

Overall, as anticipated, a higher precursor concentration under a constant total flow resulted in a thicker coating on the surface, highlighting the impact of precursor concentration on both coating thickness and growth rate. Notably, the higher precursor concentration exhibited a slower reduction in growth rate.

## 4. Discussion

The findings of this study suggest that the model ketone molecule precursor concentration has a more significant impact on the growth mode than treatment time. Several factors contribute to this observation, which we will discuss in this paper. One crucial aspect to consider is the input power, as altering the number of precursor molecules in the discharge while keeping the power constant modifies the available energy per molecule. Increasing the concentration of the organic precursor under the same operating conditions reduces the available energy for the excitation process, limiting fragmentation. This results in a softer plasma polymerization of the precursor with a limited incorporation of carrier gas atoms within the plasma-deposited coating structure.

However, when the precursor concentration is lower (below 15% in this study), the effect of treatment time becomes more evident. In such conditions, nitrogen is more involved in the chemical reactions within the plasma discharge, as indicated by the formation of amide groups in the deposited coating (Figure 7). In such circumstances, the plasma deposition process resembles a step-growth polymerization, as suggested by Yasuda et al. [55,56]. In a step-growth polymerization process, the components of the reaction mixture in the discharge change over time, affecting the growth mechanisms of the coating. In contrast, in chain-growth polymerization (i.e., soft polymerization), time has no effect on the structure of the components of the reaction mixture, with this structure remaining close to that of the original precursor molecule. For example, when the precursor concentration is high, such as at 40%, the growth mechanism could simulate chain-growth polymerization with low molecular fragmentation, unaffected by treatment time.

This raises questions about the stability of coatings deposited with various precursor concentrations. The preliminary experiments (not shown) involved placing the samples in a water bath and rubbing them with a sponge under an applied pressure of 100 g at a rubbing speed of 34 m/min for 37 cycles (a strong abrasive test). These experiments demonstrated that coatings made with a lower precursor concentration (1%) were more likely to remain on the fluoropolymer surface, possibly because they were already thicker at the start of the abrasion experiments. All other experimental conditions being identical, XPS data showed no fluorine on ETFE plasma coated with a 1% precursor concentration, while 7% of fluorine was detected when using 60% of the precursor in the plasma discharge. After the abrasion tests, 24% and 30% fluorine were found on fluoropolymer samples coated with 1% and 60% of precursor in the discharge, respectively. Moreover, the fact that more crosslinking is observed at low precursor concentrations (as more power is available per precursor molecule) and the different chemistries of the coatings at low and high precursor concentrations (containing more amide and cyano groups at low precursor concentrations) may also indicate that more plasma-deposited polymer remains on the fluoropolymer surface when a low precursor concentration is injected into the discharge.

By maintaining the plasma power per unit of monomer at a low level, it becomes feasible to deposit coatings under “soft plasma conditions” that resemble the power-deficient regime elucidated by Yasuda in vacuum polymerization systems [25,30,56,57]. According to Herbert et al. [25], using a low discharge power and a high precursor amount in aerosol-assisted deposition led to minimal fragmentation of precursor molecules and better retention of precursor molecule structure on the surface. Specifically, the authors investigated the deposition of a coating with 1H, 1H, 2H, and 2H-heptadecafluorodecylacrylate and found that a low-specific-energy discharge (point-to-plane corona discharge) and aerosol allowed for the deposition of a coating with minimal precursor molecule fragmentation and high retention of the perfluorinated chain. In a follow-up study, the effect of the physical state of the (perfluoro-1-decene) precursor was examined concerning its fragmentation in the discharge and subsequent coating properties. The retention of the precursor structure was observed to be high for both states due to the low discharge power employed in the study [57]. In another work, Da Ponte et al. [58] used a DBD system to deposit a PLA-like film and found that the chemical composition of the film can be influenced by adjusting the amount of carrier gas and aerosol precursor fed into the discharge. Increasing the carrier gas amount reduced the C1 components related to the PLA chemical composition, likely due to increased fragmentation of the lactic acid monomer in the discharge. On the other hand, a lower amount of carrier gas compared to the amount of aerosol fed resulted in higher retention of the monomer’s structure, resulting in a chemical composition of the film comparable to that of conventional PLA. Also, in another work, Profili et al. [10] confirmed that increasing the precursor concentration led to a thin-film coating with low fragmentation. In addition, electrical measurements showcased a filamentary discharge regime marked by short current pulses at various precursor concentrations. However, a precursor concentration of 40% resulted in intensified current spikes, reduced charge amplitudes in Lissajous figures, and a delayed breakdown. This emphasizes that greater precursor injection can destabilize the discharge. The literature indicates that the inclusion of precursors capable of quenching the 6.2 eV metastable state consistently leads to discharge destabilization owing to insufficient energy levels for reactive component ionization. Studies have shown that even small additions of quenchers, such as O_2_ or NH_3_, can significantly restrict the minimum frequency required for TDBD to occur [11].

Furthermore, previous results have demonstrated that a combination of a low precursor concentration (e.g., 3%) and high power (e.g., 0.9 W/cm^2^) could result in the formation of C≡N on the surface [48]. The formation of C≡N can also be influenced by parameters such as total flow, precursor concentration, and treatment time. Surprisingly, a higher total flow rate did not significantly affect the formation of nitrile groups, despite the increase in nitrogen availability in the discharge resulting from a higher total flow or a lower precursor concentration. Our research findings suggest that precursor concentration has a more prominent effect on nitrile formation, independent of the gas residence time (total flow). Indeed, the nitrile bond ceased to appear beyond a 3% precursor concentration for both total flow rates. However, reducing the precursor concentration led to an increase in the power available per molecule for fragmenting organic molecules and forming C≡N.

The formation of the C≡N bond requires approximately 891 kJ/mol of energy, which is higher than any of the other chemical bonds formed within the plasma-deposited polymer. For instance, the C≡N and C–O bonds have bond energies of 305 and 356 kJ/mol, significantly less than that of the triple bond formed between carbon and nitrogen in a nitrile group. Therefore, increased energy accessibility per molecule may promote involvement in high-energy chemical bond formation. This explains why the C≡N formation was limited to precursor concentrations lower than 3% for both total flows of 1 SLM and 5 SLM (Figure 8). Siliprandi and coworkers [59] studied thin-film deposition from HMDSO in an atmospheric pressure plasma DBD system using nitrogen as a carrier gas. The results showed the existence of C≡N in the infrared spectrum of the thin film between 2000 and 2170 cm^−1^. Their study demonstrated that the peak intensity associated with C≡N dropped significantly as the concentration of HDMSO increased from 0.5% to 1.2% inside the discharge, clearly indicating that the precursor concentration affects the amount of C≡N in the coating.

It should be noted that the intensity of the infrared feature may also be influenced by the coating thickness, which, as demonstrated in this study (Figure 10), is also affected by the plasma experimental parameters.

Moreover, a longer treatment time might affect the temperature in the discharge, resulting in a stronger fragmentation process. As Guerrero et al. [60] mentioned, in a gas discharge, the metastable states may be highly populated. Due to their extended lifetimes, they can store energy for a long period and eventually release it through different channels. Metastable species may be able to store sufficient energy to directly ionize the gas phase via Penning or associative ionization. If the metastable states easily release their energy into the background gas, they can contribute to a heating process. This phenomenon occurs over time and might produce a stronger fragmentation process leading to the formation of a more intense nitrile peak.

Additionally, the impact of gas residence time on functionalization and coating thickness over time was an interesting observation. As described, an increase in total flow from 1 SLM to 5 SLM resulted in a decrease in the Ƭr (i.e., gas residence time) within the discharge from 8.5 to 1.7 ms for a fixed amount of precursor. A longer Ƭr facilitated the ability of the molecule to remain within the discharge, react, and efficiently deposit on the surface via a combination of nitrogen and precursor molecules, a finding that is in agreement with the power/precursor flow rate data presented in Figure 1. However, this would not be the case for shorter gas residence times wherein molecules have less of an opportunity to react in the gas phase but rather interact with the freshly deposited coating. In other words, the growth mechanisms should occur primarily at the interface for short residence times rather than in the gas phase [61].

Notably, the growth rate data indicated a slower deposition for a shorter gas residence time, as reported by Zajíčková et al. [31]. In their work, Da Ponte et al. [58] also mentioned that increasing the carrier gas fed into the discharge from 4 SLM to 5 SLM while keeping the aerosol amount constant (lactic acid) resulted in a decrease in the deposition rate from 10 nm/min to 8.7 nm/min.

Concerning coating thickness, the data from the present study suggest that more than one growth mechanism should be considered to explain the observed behavior. The initial layer of the coating adheres to the top of the ETFE structure and functionalizes its surface based on reactions with the radicals formed right after plasma ignition. This explains why, for all the investigated experimental conditions, the deposition rates were almost identical (60 nm/min) at the beginning of the treatment. On the contrary, longer treatment times led to a decrease in the growth rate until a plateau was reached.

Changes in substrate temperature likely played a role in our case, as the polymer became hot enough to be affected by temperature, as pointed out by Destrieux et al. [43]. However, the decrease in the growth rate and the creation of a plateau over time indicate that the formation of the coating could involve multiple mechanisms. It is possible to argue that while material was being added from the plasma process, energetic particle bombardment and the etching of weaker areas also occurred, leading to the partial elimination of the coating from the uppermost layers. Heggman noted that in plasma polymerization processes, excess energy conditions allow for a wide range of endothermic chemical reactions to take place [62].

As stated previously, the decrease in gas residence time resulted in an increase in the amide/ketone infrared ratio due to the incorporation of more nitrogen. From a chemical point of view, amide groups are known to be less prone to promoting electrophilic reactions than ketone moieties [53]. Therefore, one could suggest that the coating deposition rate on a layer containing more amide groups may be slower. For instance, Yasuda et al. [63] stated that the deposition rate could increase due to the additional effect of oxygen-containing moieties on the surface.

## 5. Conclusions

In this study, a fluoropolymer was coated with an organic thin-layer coating using a nitrogen atmospheric pressure plasma DBD at different precursor concentrations and different gas residence times in static mode for various durations.

The XPS results showed that a higher precursor concentration in the discharge limits molecule fragmentation, resulting in lower nitrogen content in terms of (C–N, C–O)/(C–C, C–H) formation on the surface. This finding was supported by FTIR analysis, particularly in the 1500–1800 cm^−1^ range, where the behavior of the C=O bonds as a function of the precursor concentration provided an explanation of the fragmentation process of the model ketone organic precursor molecule. Increasing the precursor concentration in the discharge resulted in an amide/ketone ratio reduction in the coating. At constant power, increasing the precursor concentration reduces the amount of power available per molecule for molecular fragmentation, leading to a softer plasma polymerization of the precursor and a lower amide/ketone ratio. The highest amount of nitrogen incorporated into the coating was observed at a 1% precursor concentration. In this case, the combination of constant power (0.75 W·cm^−2^) and a low precursor concentration resulted in the efficient fragmentation of the precursor molecules and the incorporation of the nitrogen carrier gas atoms within the coating structure.

Under these plasma operating conditions, in addition to the high contribution of amides to the carbonyl peak, the stretching vibration of C≡N groups at 2160 cm^−1^ could be detected in the plasma-deposited coating, further highlighting the incorporation of nitrogen in the coating. It was also proven that the precursor concentration had a stronger impact on the amide/ketone ratio evolution compared to the treatment time. In fact, the treatment time only showed a marginal effect on the formation of amides/ketone for precursor concentrations below 15%.

Moreover, the residence time of the gas in the plasma region was also shown to be important in the thin-film growth mode investigation. Increasing the gas residence time in the plasma zone increased the incorporation of nitrogen in the amide form into the coating. The coating thickness was also affected by the gas residence time in the discharge, wherein the coating thickness increased with the gas residence time. In addition, an increase in coating thickness was also obtained when subjecting the substrate to a plasma discharge for longer treatment periods. In this study, a fluoropolymer was subjected to coating with an organic thin layer using a nitrogen atmospheric pressure plasma dielectric barrier discharge (DBD) at different precursor concentrations and various gas residence times in static mode for different durations.

The XPS results revealed that higher precursor concentrations in the discharge limit molecular fragmentation, resulting in lower nitrogen content in terms of (C–N, C–O)/(C–C, C–H) formation on the surface. This observation was supported by FTIR analysis, particularly in the 1500–1800 cm^−1^ range, where the behavior of C=O bonds, as a function of precursor concentration, helped to elucidate the fragmentation process of the model ketone organic precursor molecule. Increasing the precursor concentration in the discharge led to a reduction in the amide/ketone ratio in the coating. At a constant power of 0.75 W/cm^−2^, increasing the precursor concentration decreased the available power per molecule for molecular fragmentation, resulting in a softer plasma polymerization of the precursor and a lower amide/ketone ratio. The highest amount of nitrogen incorporated into the coating was observed at a 1% precursor concentration. In this case, the combination of constant power and a low precursor concentration facilitated the efficient fragmentation of the precursor molecules and the incorporation of nitrogen carrier gas atoms within the coating structure.

Under these plasma operating conditions, in addition to the significant contribution from amides to the carbonyl peak, the stretching vibration of C≡N groups at 2160 cm^−1^ could be detected in the plasma-deposited coating, further emphasizing the incorporation of nitrogen into the coating. It was also demonstrated that the precursor concentration had a more substantial impact on the evolution of the amide/ketone ratio compared to treatment time. In fact, treatment time exhibited only a marginal effect on the formation of amide/ketone for precursor concentrations below 15%.

Furthermore, the residence time of the gas in the plasma region was shown to be crucial in investigating the growth mode of the thin film. Increasing the gas residence time in the plasma zone enhanced the incorporation of nitrogen in the amide form into the coating. Coating thickness was also influenced by the gas residence time in the discharge, wherein an increase in coating thickness occurred with longer gas residence times. Additionally, an increase in coating thickness was observed when subjecting the substrate to a plasma discharge for longer treatment times.

## Figures and Tables

**Table 1:** The precursor amount (g·h^−1^) per various precursors (%) (e.g., 1, 3, 5, 10, 15, and 40%) in 1 and 5 SLM total flows.

	Total Flow (SLM)							
	1	5	1	5	1	5	1	5
Precursor (%)	Gas Residence Time (Ƭr, ms)		Precursor Amount (g·h^−1^)		Nitrogen Amount (SLM)		Power/Precursor Flow Rate (W/g·h^−1^)	
1	8.5	1.7	1.2	6	0.99	4.99	18.6	3.7
3	8.5	1.7	3.6	18	0.97	4.97	6.2	1.25
5	8.5	1.7	6	30	0.95	4.95	2.8	0.75
10	8.5	1.7	-	60	-	4.9	-	0.39
15	8.5	1.7	18	90	0.85	4.85	1.2	0.24
40	8.5	-	48	-	0.6	-	0.46	-

## Data Availability

Data are contained within the article.
